# Structure and Function of the Nuclear Receptor Superfamily and Current Targeted Therapies of Prostate Cancer

**DOI:** 10.3390/cancers11121852

**Published:** 2019-11-23

**Authors:** Baylee A. Porter, Maria A. Ortiz, Gennady Bratslavsky, Leszek Kotula

**Affiliations:** 1 Department of Urology, Upstate Cancer Center, SUNY Upstate Medical University, Syracuse, NY 13210, USA; porterba@upstate.edu (B.A.P.); ortizma@upstate.edu (M.A.O.); bratslag@upstate.edu (G.B.); 2 Department of Biochemistry and Molecular Biology, SUNY Upstate Medical University, Syracuse, NY 13210, USA

**Keywords:** nuclear receptors, androgen receptor, prostate cancer, STAT3, treatment, transcription factors

## Abstract

The nuclear receptor superfamily comprises a large group of proteins with functions essential for cell signaling, survival, and proliferation. There are multiple distinctions between nuclear superfamily classes defined by hallmark differences in function, ligand binding, tissue specificity, and DNA binding. In this review, we utilize the initial classification system, which defines subfamilies based on structure and functional difference. The defining feature of the nuclear receptor superfamily is that these proteins function as transcription factors. The loss of transcriptional regulation or gain of functioning of these receptors is a hallmark in numerous diseases. For example, in prostate cancer, the androgen receptor is a primary target for current prostate cancer therapies. Targeted cancer therapies for nuclear hormone receptors have been more feasible to develop than others due to the ligand availability and cell permeability of hormones. To better target these receptors, it is critical to understand their structural and functional regulation. Given that late-stage cancers often develop hormone insensitivity, we will explore the strengths and pitfalls of targeting other transcription factors outside of the nuclear receptor superfamily such as the signal transducer and activator of transcription (STAT).

## 1. Introduction

The nuclear receptor superfamily is a family of transcription factors that are widely expressed throughout the body. This family functions in well-organized signaling pathways that heavily rely on tissue microenvironment and when disrupted, endogenously or exogenously, can cause organ dysfunction, cancer, or loss of tissue integrity. Pharmacological intervention inhibiting signaling pathways of members of this family has been used for treatment of many diseases. Based on the evolution and robust treatment response to anti-androgen therapies, we examine different agents currently used in different stages of prostate cancer progression as well as new targets being explored due to a rise in treatment resistance. 

**Figure 1:**
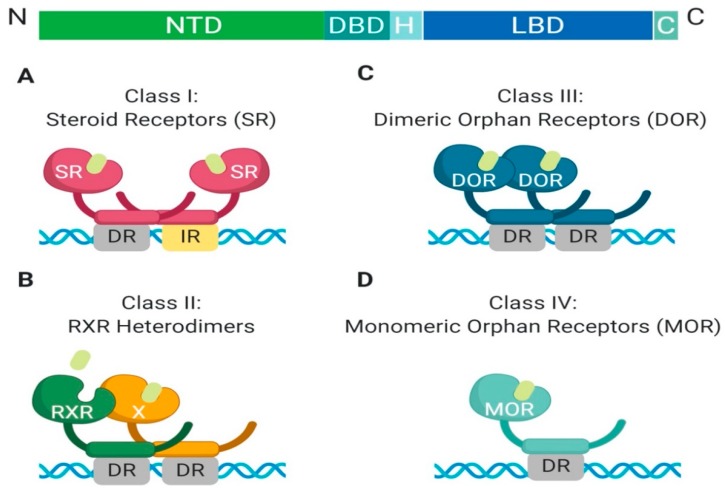
**Schematic illustration of the classical nuclear receptor superfamily**. (**A**–**D**) graphically represent the four classes of the nuclear receptor superfamily which are defined based on dimerization (homo, hetero, or mono), DNA binding (direct repeat or inverted repeat), and ligand specificity (required, or not required). Class I, Steroid Receptor (also known as nuclear hormone receptors); Class II, RXR Heterodimers; Class III, Dimeric Orphan Receptors; Class IV, Monomeric Orphan Receptors. *Abbreviations: NTD, N-terminal domain; DBD, DNA-binding domain; H, Hinge region; LBD, Ligand-binding domain; C, Variable C-terminus; DR, Direct Repeat; IR, Inverted Repeat.*

The nuclear receptor superfamily is comprised of over 500 members. This superfamily is further divided into four classes based on key characteristics such as dimerization, DNA binding motifs and specificity, and ligand binding. The four classes include steroid Receptors (Class I), RXR heterodimers (Class II), homodimeric orphan receptors (Class III), and monomeric orphan receptors (Class IV). Although there are some significant structural and functional differences between the classes, some key structural components are preserved, which are permissive to their respective functions (Figure 1) [1].

All nuclear receptor superfamily members contain a variable N-terminal domain (NTD), a DNA binding domain (DBD), a hinge region, a conserved ligand-binding domain (LBD), and a variable C-terminal domain. The two most highly conserved domains amongst all nuclear receptors are the DNA binding domain and the ligand-binding domain. The DNA binding domain contains two zinc finger motifs, which act as a hook, that allow binding to chromatin within the nucleus [2]. Each class has different DNA binding recognition sequences, which range from variable half-sites with inverted repeats, direct repeats, or no repeats within the DNA sequence [1].

The ligand-binding domain of nuclear receptors remains highly conserved in function but differs in specificity and affinity to specific ligands [1,3]. All classes, excluding orphan receptors, are ligand-activated. Ligand binding at the LBD induces an allosteric change, inducing activation [1,3]. Ligands within each class of nuclear receptors have similar structures. Furthermore, classification of the ligand determines which class of nuclear receptors each belongs to [1,3]. For example, endogenously expressed ligands for these receptors can be hormones, metabolites, or enzymatic ligands, as well as unidentified ligands [1,3].

Another feature which differentiates class members is partner dimerization within the nucleus. Classes I–III require dimerization while Class IV does not. Additionally, Class I and III require homodimerization, which can provide stronger zinc finger binding to DNA, while Class II requires heterodimerization [1].

There have been modifications to each subclass based on new information gathered through structural analysis and sequencing data. For this review we will focus on the classical subdivisions of the nuclear receptor superfamily defined by the hallmarks of nuclear receptor superfamily structure and function such as dimerization, DNA binding motifs and specificity, and ligand-binding activation.

### 1.1. Class I: Overview of Steroid Hormone Receptors, Structure and Function

**Figure 2:**
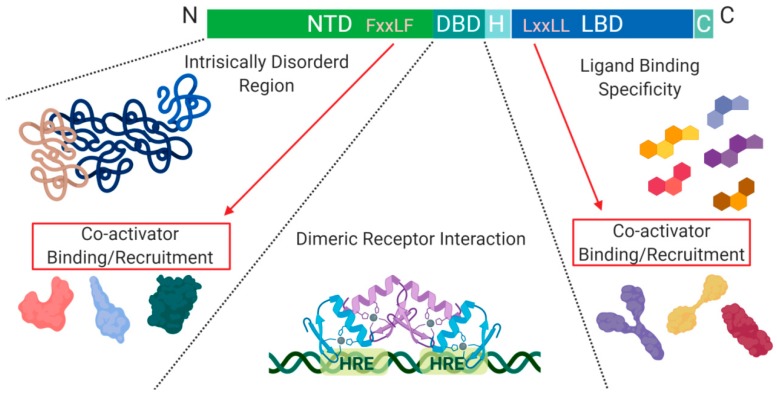
**Schematic illustration of the nuclear receptor hormone family.** Structural differences within the nuclear receptor hormone family occur at the NTD, DBD, and LBD. The functional differences are defined based on co-activator recruitment, dimeric receptor interactions, and ligand binding. *Abbreviations: NTD, N-terminal domain; DBD, DNA-binding domain; H, Hinge region; LBD, Ligand-binding domain; C, Variable C-terminus; HRE, Hormone Response Elements.*

All members of Class I are grouped based on shared characteristics and functions (Figure 2). First, they are ligand-activated receptors, for ligand-binding induces a conformational change that allows for homodimerization and subsequent DNA-binding. Additionally, Class I members have a unique role in the maintenance of cellular homeostasis, gene expression regulation in embryogenesis, and tissue development, as well as their ability to respond to extracellular signals in an endocrine manner, which allows the cells to adapt to systemic environmental changes. Within Class I nuclear hormone receptors, there can be redundancy of individual members to perform each other’s transcriptional functions, but it is highly dependent on tissue-specific expression of endogenous ligands [4].

Another level of regulation, which has been best characterized for Class I nuclear receptors, is determined by the presence of co-activators and/or repressors within the nucleus. These can be specific to a particular receptor within the class and are critical for transcription initiation or repression [4].

The DNA binding domain (DBD), is a cysteine-rich domain that has a conserved amino acid sequence and encodes two zinc (Zn) finger motifs. Specifically, C1 to C4 are responsible for the first zinc finger motif, and C5 to C8 are responsible for forming the second. Each finger motif then chelates a Zn(II) ion, allowing for a structural DNA recognition site to form [2]. The zinc finger motifs are known as the P-box and D-box, where the P-box refers to the 1st zinc finger motif in the sequence, which directly interacts with DNA, while the 2nd zinc finger site does not [5]. The half site sequences on DNA that allow for zinc finger binding are highly conserved, as seen in the estrogen and glucocorticoid receptors. This level of similarity leads to single nucleotide or amino acid mutations of a zinc finger domain to cause receptor protein promiscuity. Wherein, receptor proteins can recognize hormone response elements of other receptors on DNA, and initiate transcription of genes non-specific to the external signal received [2]. Additionally, the hexameric half-sites recognized by zinc finger motifs for the androgen receptor, progesterone receptor, and glucocorticoid receptor are highly conserved [5]. However, the specific difference that allows for response element specificity is how the zinc fingers interact with each other once a dimer is formed, either head-to-head or head-to-tail [6].

Another conserved structural feature among Class I nuclear receptors is their ligand-binding domain (LBD). The LBD contains around 12 α-helices, three of which form the hydrophobic pocket, also known as the ligand-binding pocket (LBP). The ligand binding specificity within the pocket is determined by conformational differences which cause steric hindrance of non-specific ligands [7].

A structural feature of nuclear hormone receptors that has previously been overlooked is the activation function-1 (AF-1) protein domain within the N-terminal region and the activation function-2 (AF-2) in the LBD. This is a common feature found in Class I–III, but not in Class IV [8]. The presence of AF-1 in the intrinsically disordered region of the N-terminal allows for flexibility and becomes ordered when bound to individual partners [8]. On the C-terminal end, AF-2 requires ligand-binding to become active but remains ordered in all states [8]. Unfortunately, when AF-2 is spliced out, the protein can undergo a gain of function mutations that no longer require ligand-binding for activation and cause dysregulated protein expression [8]. On the outside of the AF-2 protein domain, at α-helix 12, there is a hot spot for steroid co-activator binding (SRC) mediated through its LxxLL motif, which promotes transcriptional activity (Figure 2) [9]. Similarly, within the NTD, there is a five amino acid long motif FxxLF that binds and stabilizes the N-terminal and C-terminal domains. This binding promotes the stabilization of dimers through an active conformational state, preventing ligand-bound dissociation [5,9,10].

The N-terminal and C-terminal of the nuclear hormone receptors are crucial for the recruitment of co-activators within the nucleus, which can vary significantly between family members [11]. The variability between nuclear hormone receptor co-activator binding is most likely caused by the specific amino acid arrangement in the NTD rather than difference in the chemical characteristics of amino acids present [12]. Binding of the NTD with its preferred co-activator results in a highly coiled structure, which can alter the structural properties of the receptor. For example, the androgen receptor, in this highly coiled-state, becomes highly resistant to proteases [12].

Overall, nuclear hormone receptors play a crucial role in body homeostasis. Thus, mutations, misfolding, or alteration of signaling pathways can often lead to systemic organ dysfunction. Each nuclear hormone receptor has a specific set of target genes, which display tissue-specificity, under a ligand-activated state initiated by a specific ligand.

#### 1.1.1. Class I: The Androgen Receptor, Structural and Functional Differences

The androgen receptor (AR) is essential for male sexual differentiation, bone growth, muscle homeostasis, and development [5]. AR is activated when α-dihydrotestosterone (DHT) binds to the LBP within the LBD of AR, inducing a conformational change. This leads to the activation of AR through the disassembly of chaperone proteins such as HSP70 and HSP90 and simultaneous exposure of a nuclear localization signal (NLS) in the DBD [5,13].

Unlike other nuclear hormone receptors, androgen response elements have high specificity and low-affinity interactions with DNA [5]. The literature suggests AR requires increased stability to bind DNA at specific androgen response element sites through head-to-head zinc finger dimerization [5]. Additionally, AR has increased specificity to DNA recognition sites by recognizing both an inverted repeat and a direct repeat known as ADR3 [6]. Comparatively, the glucocorticoid receptor (GR) has been shown to have less bulky amino acids in the zinc finger motifs which form an open pocket within the head-to-head zinc dimer [6]. The AR contains amino acids that allow for a more compact structural conformation which reduces pocket size, ultimately increases homodimer stability [5,6,12].

The NTD of AR fosters a plethora of protein–protein interactions due to the variability in poly-glutamine and poly-glycine length which contributes to its highly disordered nature [5,12]. The variability in glycine and glutamine residue repeats in the NTD of AR allow for interaction with numerous binding partners due to increased flexibility, increased number of conformations, and modified functionality [5,14]. A decreased amount of glutamine and glycine repeats increases the transcriptional activity of AR most likely due to decreased protein–protein interactions with co-repressor binding partners [5,12].

**Figure 3:**
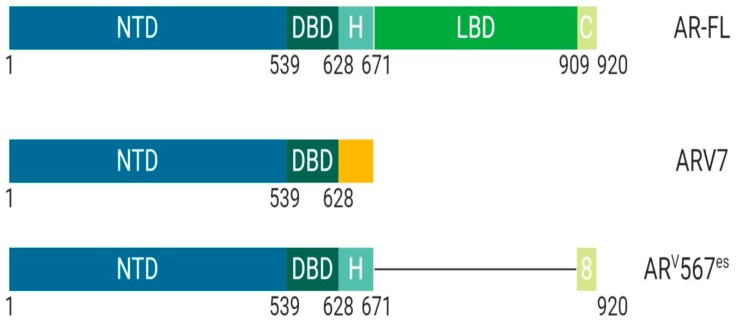
**Schematic illustration of androgen receptor splice variants**. The most common AR splice variants implicated in prostate cancer treatment resistance are ARV7 and AR^V^567^es^. Splice Variant ARV7 lacks the LBD and therefore does not require ligand binding for activation which allows for constitutive activity in the context of low ligand availability during PCa treatments; ARV7 can also bind AR-full length in the nucleus, promoting continuous transcriptional activity. Splice variant AR^V^567^es^ has an exon skipping mutation for exons 567 but retains exon 8 and therefore can bind DNA and remain constitutively active without ligand activation. However, the remaining exon 8 can potentially allow for co-activator recruitment and various conformational differences independent of AR-full length and ARV7. *Abbreviations: AR, Androgen receptor; NTD, N-terminal domain; DBD, DNA-binding domain; H, Hinge region; LBD, Ligand-binding domain; C, Variable C-terminus.*

Upregulation of AR splice variants is commonly observed in different malignancies:

ARV7 is a splice variant of AR commonly upregulated after androgen deprivation therapy (Figure 3). ARV7 lacks an LBD and does not require a ligand for active transcription. Recent studies have shown that ARV7 can homodimerize with full length-AR and repress the transcription of tumor suppressor genes [15]. The ARV7 splice variant is constitutively active and requires full length-AR to repress transcription [15].

ARv567es is a splice variant of AR that lacks exons 5, 6, and 7 while retaining exon 8 and does not require ligand binding for transcriptional activity (Figure 3) [16]. The splice variant ARV567es requires homodimerization with full length-AR for actively transcribing target genes. Unique from other forms of AR, this splice variant localizes to the nucleus wherein it waits for full length-AR to begin any activity [16]. Recent studies identify that ARv567es actively transcribes a unique set of target genes that are distinct from full length-AR, indicating that expression of ARv567es could be a fail-safe mechanism used by tumor cells to promote cell survival [16].

#### 1.1.2. Class I: The Progesterone Receptor, Structural and Functional Differences

**Figure 4:**
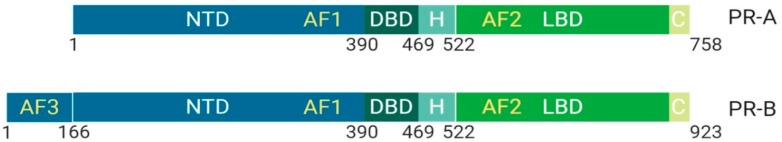
**Schematic illustration of progesterone receptor isoforms.** The progesterone receptor has two distinct isoforms, PR-A and PR-B. PR-B is more transcriptionally active due to the extended N-terminal domain that contains AF3 (unique domain), an activation function unit that allows for an increase in co-binding partners and activity. The isoform PR-A is less transcriptionally active than PR-B and primarily functions in non-genomic pathway activity. Its upregulation is implicated in cancers. *Abbreviations: PR, Progesterone receptor; NTD, N-terminal domain; DBD, DNA-binding domain; H, Hinge region; LBD, Ligand-binding domain; C, Variable C-terminus; AF1, Activation function-1; AF2, Activation function-2; AF3, Activation function-3.*

The ligand for the progesterone receptor (PR) is progesterone. Ligand-activation of PR plays a critical role in female mammary gland development/homeostasis and other female reproductive organs [10]. Additionally, PR is expressed in prostate tissue and plays an essential role in tissue microenvironment and stromal cell differentiation [17]. PR has two isoforms which have differential roles in normal organ functioning and the balanced expression of both isoforms is critical for normal tissue function [10,18]. The two isoforms—Progesterone Receptor-A (PR-A) and Progesterone Receptor-B (PR-B)—differ in size at the NTD (Figure 4) [10].

PR-B has an extended NTD, which is called a unique domain or activating function domain 3 (AF-3) [10]. PR-B is more transcriptionally active than PR-A and plays a crucial role in mammary development. However, dominant expression of PR-A is implicated in cancer onset due to non-genomic activities mediated through the binding of activated Src kinases [10].

Post-translational modifications of the hinge region of PR-A/B allows the PR to interact with chromatin-associated high mobility proteins 1/2 (HMGB1/2) and Jun dimerization protein 2 (Jun2) [19,20]. These interactions have been shown to increase transcriptional activity of PR [20]. HMGB1/2 is a protein that increases DNA-protein binding interactions indirectly through increasing the number of contacts of the PR to DNA through dynamic conformational change [21,22,23].

Furthermore, recent evidence suggests a role of PR in prostate cancer progression, specifically through its role in regulating smooth actin muscle (SMAα) in stromal cells [17,24]. PR levels were upregulated after castration in patients with prostate cancer, which could contribute to treatment resistance due to its role in stromal cell differentiation and mobility [17,24]. Until recently, the role of PR in prostate cancer was not evaluated but maybe a key player in understanding current mechanisms of treatment resistance.

#### 1.1.3. Class I: The Estrogen Receptor, Structural and Functional Differences

**Figure 5:**
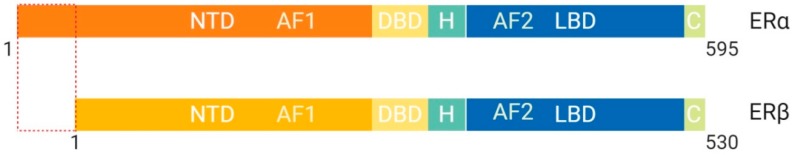
**Schematic illustration of estrogen receptor isoforms.** The estrogen receptor has two isoforms, ERα and ERβ, and is comparatively smaller than other family members. The isoform ERα has an extended NTD which allows for more transcriptional activation, whereas, ERβ has a shorter NTD and is less transcriptionally active. Most often the ERα isoform is upregulated in cancers. *Abbreviations: ER, Estrogen receptor; NTD, N-terminal domain; DBD, DNA-binding domain; H, Hinge region; LBD, Ligand-binding domain; C, Variable C-terminus; AF1, Activation function-1; AF2, Activation function-2.*

The estrogen receptor (ER), whose ligand is estradiol, plays a key role in female reproduction [18]. There are two isoforms of the ER known as ERα and ERβ, which are structurally distinct and perform different functions [18] (Figure 5).

These structural differences allow for interaction with different binding partners and subsequent transcriptional activation of distinct target genes [4]. ERβ protein lacks an AF-1 region, which suggests that ligand binding is essential for function through LxxLL motif binding at AF-2 α-helix 12 [4]. ERα protein contains both AF-1 and AF-2 structural features and is more transcriptionally active than ERβ [4]. Previous studies have shown that the ER binds multiple estrogen response elements (ERE) that vary based on the second didactic half-site [4]. It is suggested that the variability of ERE allows for modulation of allosteric regulation and ultimately, co-activator recruitment [4]. Post-translational modifications of ERα such as methylation of Arg^260^ by protein arginine methyltransferase (PMT1) are necessary for ERα to interact with the p85 subunit of PI3K and c-SRC [25]. On the other hand, acetylation of Lys^266/268^ by p300 enhances the transcriptional activity of ERα by increasing ERE binding specificity [26].

Recent evidence suggests that ERβ plays an essential role in the proliferation of prostatic epithelial cells, which is a feature of prostate cancer progression [27]. The downregulation of ERβ as a consequence of current prostate cancer treatments could increase the proliferation of prostatic epithelial cells and contribute to disease progression. Furthermore, studies show that a subset of patients with high-grade tumors had a loss of the ERβ gene locus [27]. Similarly, mice that had a loss of ERβ developed prostate cancer, which could be a useful biomarker for early detection of prostate cancer [27].

ER has a pivotal role in the female reproductive system and secondary sexual characteristic development and function [4,28]. ER has been well characterized as a key player in breast cancer development. The differential expression of ER’s isoforms has been implicated in breast cancer metastasis, and can be used to determine treatment, prognosis, and stage of the disease [29]. Similar to prostate cancer, some forms of breast cancer are also hormone-sensitive, with approximately 70% of them being hormone-sensitive and ER positive [30]. Based on the currently available targeted therapies for breast cancer (also referred to as Endocrine Therapy (ET)) and their superiority to chemotherapy with regards to tolerance, efficacy, and less severe side effects, breast cancer is subdivided into distinct biologic groups based on receptor expression: Estrogen Receptor (ER+), Progesterone Receptor (PR+), those that express the epidermal growth factor receptor 2 (HER2+), and those that do not express either are classified as triple negative BC [30].

Production of estrogen in females is analogous to testosterone production in males. This allows for some of the same agents used for chemical castration to be used for ovarian ablation (see below). The use of LHRH analogs allows for the downregulation of estrogen production by the ovaries, the main source of estrogen in pre-menopausal women. Aromatase inhibitors (i.e., anastrozole, exemestane and letrozole) inhibit the enzyme aromatase, which converts androgens into estrogens in tissues outside of the ovaries. This therapy works best in post-menopausal women, since production of estrogen post-menopause is not in the ovaries [30].

#### 1.1.4. Class I: The Glucocorticoid Receptor, Structural and Functional Differences

The ligands for the glucocorticoid receptor (GR) are glucocorticoids which are produced by the adrenal cortex. This protein is ubiquitously expressed throughout the body and mediates metabolism as well as anti-inflammatory response through ligand-binding activation of cortisol. Recent studies have shown that specificity of DNA binding for GR is mediated by amino acid composition in the DBD of the protein which most likely contributes to the high level of protein expression throughout the entire body but lack of overlapping function with family members such as AR [31]. More specifically, one amino acid polymorphism will allow for a different conformation and therefore lead to different target gene binding [31]. However, mutation of the DNA binding domain could also decrease DNA binding specificity and allow for activation of target genes that belong to other family members such as AR [31]. Furthermore, GR, unlike other nuclear hormone receptors, has an abundance of acidic residues in the NTD, which increase its interaction with co-activator proteins [32]. Additionally, the AF-1 in the NTD of GR can perform 65% of normal functioning compared to wildtype GR [32]. Most other nuclear receptor family members require both AF-1 and AF-2 for proper function. However, the modification of acidic residues in the NTD allows for GR to function in the absence of a ligand [32].

More recent evidence suggests that TIF2.0 (p160 co-activator TIF2) directly interacts with the NTD of GR [33,34]. Previously, TIF2 has been shown to solely interact with the LBD of other nuclear hormone receptors wherein, TIF2.0 has an extended NTD [34]. Through NTD binding of TIF2.0 to GR, a conformational change occurs, allowing for an increased α-helix formation [33]. Similarly, binding of TIF2.0-GR was shown to inhibit co-repressor binding, which suggests a unique mechanism of increased transcriptional activity of GR [33].

### 1.2. Class II: RXR Heterodimers, Structure and Function

The Retinoid X Receptor (RXR) ligand is 9-cis-retinoic acid or alitretinoin, which plays a role in lipid metabolism, apoptosis, and the immune system [35]. RXR unlike nuclear hormone receptors, are highly promiscuous with regards to their binding partners [3]. The key feature of Class II nuclear receptors is that RXR dimerization is required for activation. The RXR can bind to itself and promote activation, but other members of this family such as Peroxisome Proliferator Activator Receptor (PPAR), Pregnane X Receptor, and Liver X Receptor all require heterodimerization with RXR to translocate to the nucleus [3]. All receptors in Class II bind to unique response elements, which makes RXR dynamic and heavily relied upon for normal physiological function [36]. Downregulation or loss of RXR signaling has been shown to promote inflammation of vital organ systems such as the liver [37]. Interestingly, RXR without ligand binding can still bind DNA and perform functions such as recruiting co-repressor complexes to repress gene expression through heterodimerization with Retinoic Acid Receptor (RAR) [38]. In cancer cells, RXR is sequestered in the cytoplasm by the co-repressor complex AEG-1/MTDH/LYRIC which decreases its transcriptional activity [38].

### 1.3. Class III: Homodimeric Orphan Receptors, Structure and Function

Homodimeric orphan receptors are different from Class I and II in that no ligand has been identified for their activation [1,3]. Homodimeric orphan receptors are structurally similar to other family members but differ in sequence-specific binding to DNA [3]. The Class III nuclear receptor family bind to direct repeat and palindromic sequences [3]. Additionally, homodimeric orphan receptors have highly constitutive transactivation and transrepression functions, suggesting that perhaps no ligand is required for activation [3]. In some cases, Class III receptors bind similar target genes as nuclear hormone receptors and therefore, may play a critical role in alternative pathway activation [1,3]. For example, RevERbAα is an example of a homodimeric, as well as a monomeric, orphan receptor. When RevErbAα binds as a dimer to DNA, it acts as a transcriptional repressor for genes that are typically activated by Class II RXR-RAR receptors [39]. Interestingly, RORα is a Class IV, a monomeric orphan receptor that is critical for transcriptional activation of genes essential for proper cerebellar development [40,41]. A gain of function mutation at the zinc finger binding motifs of RORα allows the receptor to bind as a dimer to a subset of target genes while still maintaining its original function. However, compared to the well-characterized Class I receptors, there is a lack of functional and structural information that differentiates Class III receptors [41].

### 1.4. Class IV: Monomeric Orphan Receptors, Structure and Function

Monomeric orphan receptors are similar to Class III in that they do not require a ligand for activation. However, functionally they have a distinct role in steroid synthesis [42]. The Class IV nuclear receptor, Steroidogenesis Factor-1 (SF-1) monomerically binds to steroidogenic enzymes at the DNA enhancer sequence in all tissues responsible for steroid synthesis [3,43]. SF-1 is expressed in all steroidogenic tissues where the receptor remains constitutively active [42]. It was shown that the loss of gene expression of SF-1 results in the failure of organ development during embryogenesis [42,43]. While Class IV nuclear receptors are not directly involved in hormone signaling, this class plays a critical role throughout early sexual differentiation as well as in hormone biosynthesis [43]. Similar to Class III nuclear receptors, there is still a lot to be understood regarding the structure and function of monomeric orphan receptors.

## 2. Overview of Targeting Transcription Factors in Prostate Cancer

Transcription factors have been shown to play a key role in initiation of many cancers. Due to their role in regulating gene transcription and maintaining cellular homeostasis, the loss of their regulation results in a gain of oncogenic function and/or loss of tumor suppressors [44]. While all transcription factors share similarities in their ability to regulate gene transcription, understanding their differences in mode of activation and upstream/downstream signaling pathways becomes essential to modulate their function as potential therapeutic targets.

Our current understanding of how different transcription factor are regulated and activated, has identified different points at which these can be potentially targeted for cancer treatment, such as blocking co-activator binding, nuclear localization, upstream protein signaling, ligand-binding, or interfering with DNA binding. Furthermore, understanding differential expression of these targets in different tissues, and their function can better guide therapeutic development to reduce potential side effects. Additionally, it is important to be mindful of the effects these therapies might have in the natural course of cancer progression as it can potentially increase metastatic potential of the tumors through adaptive selection [44].

Throughout prostate cancer progression, there are unique features of protein expression and signaling pathway modifications which provide information as to which transcription factor can be effectively targeted at different stages of disease. The major signaling pathways implicated in prostate cancer such as PI3K, AKT, c-MYC, and AR give clues as to what is driving tumor growth and development [45].

To evaluate the clinical benefits and pitfalls of current prostate cancer treatments, we will focus on two differentially activated transcription factors, one of which is part of the standard of care in prostate cancer treatment (AR), and a newly identified but promising target (STAT3). AR is highly targeted in prostate cancer (PCa) at different stages of disease progression and namely earlier rather than late disease progression. There are multiple treatments available for an early, hormone-sensitive disease that have different mechanisms of action to target the AR pathway but remain non-curative due to loss of tissue-specificity, microenvironment, and alternative nuclear receptor activation. Late-stage of PCa disease progression is hormone-independent and leaves patients with few treatment options, which is why developing new treatments to target other transcription factors implicated in PCa, such as STAT3, is essential.

### 2.1. Currently Targeted Transcription Factor in Primary Prostate Cancer

Primary prostate cancer (PCa) is hormone-driven and mediated by the ligand-activated nuclear receptor and transcription factor AR. While there are different drug treatments for primary PCa, most are focused on targeting AR signaling, directly or indirectly. Androgen deprivation therapy (ADT), also known as chemical castration, is primarily used to decrease tumor size through downregulation of AR signaling. This can alleviate symptoms associated with enlargement of the prostate, such as urinary incontinence and impotence [46]. Patients that undergo ADT or prostate resection can later present with increased prostate-specific antigen (PSA) levels, referred to as biochemical recurrence. At this point, treatments targeting androgen production and signaling are used.

#### 2.1.1. Androgen Deprivation Therapy: Luteinizing Hormone-Releasing Hormone (LHRH) Agonist and Antagonist

**Figure 6:**
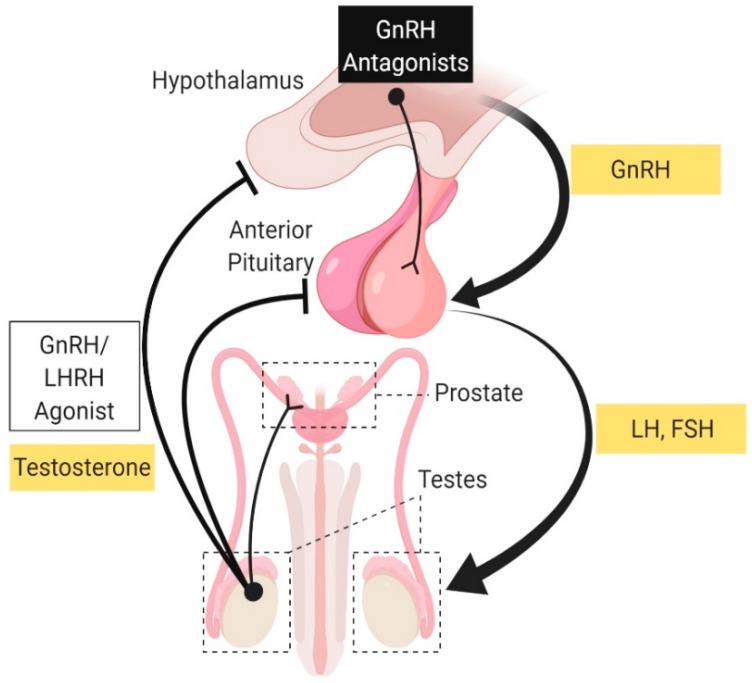
**Schematic illustration of the hypothalamic–gonadal axis (HGA):** Site of action of luteinizing hormone-releasing hormone (LHRH) agonist and Gonadotrophin Releasing Hormone (GnRH) analogs. *Abbreviations: LH, Luteinizing-Hormone; FSH, Follicle Stimulating Hormone.*

Primary prostate cancer is a hormone sensitive cancer defined by the localization of a tumor to the prostate and surrounding tissue [46]. The current standard of care for primary PCa is either radical prostatectomy, radiation, or chemical castration. Chemical castration is accomplished using a LHRH agonist. Continuous treatment with LHRH agonists causes desensitization of the gonadotrophs in the anterior pituitary, which leads to a decrease in the production of LH, with a subsequence decrease in androgen production by the testis [47]. The GnRH antagonist (aka LHRH antagonist) is a more recent drug used for chemical castration which antagonizes the production of LH from the anterior pituitary (Figure 6) [47].

LHRH agonist, as well as GnRH antagonist, are robust treatments that indirectly act on androgen signaling pathways, with an overall effect of reducing activation of AR, through decrease in androgen production.

#### 2.1.2. Anti-Androgen Therapy: Bicalutamide

Bicalutamide is an anti-androgen agent used in combination with LHRH agonists after biochemical recurrence is detected. LHRH treatment alone can lead to increased AR expression in the tumor and therefore, requires a combination of treatments in order to reduce tumor size [48]. Bicalutamide acts as an anti-androgen by abrogating co-activator recruitment through direct binding at the recruitment motif in the LBD of AR [49,50]. High selectivity of bicalutamide reduces any off-target effects that can arise from activation of other nuclear receptors by drugs with low affinity and selectivity [48]. The primary purpose of combining the use of bicalutamide and LHRH agonist is that bicalutamide has been shown to sensitize the anterior pituitary to release LH when LHRH agonists are administered [48].

The use of ADT in combination with anti-androgen therapy for PCa treatment is highly effective at shrinking tumor size for 12–24 months [51]. This treatment improves quality of life and prolongs survival of patients [51]. Combination therapy with bicalutamide and LHRH agonist allows for an extension in the effectiveness of treatment when compared to the use of either individually [51].

Unfortunately, most patients undergo biochemical recurrence within an average of 24 months of treatment. Biochemical recurrence can occur in multiple ways, including, intratumoral synthesis of androgen or reactivation of the androgen receptor [48,52]. Intratumoral synthesis of androgen can occur through the increased enzymatic activity of cytochrome P450 17A1 (CYP17A1, a variant of CYP17), CYP17A1 reduces pregnenolone, an androgen precursor, to a weak androgen, DHEA [53]. DHEA can bind full length-AR and promote gene expression [54]. Reactivation of AR can occur through multiple ways including upregulation of ARV7/AR^v^567^es^ splice variants and LBD point mutation (W741C) [15]. Upregulation of ARV7 induces transcriptional repression of tumor suppressor genes and as a result, can promote tumor regrowth [15]. Upregulation of AR^V^567^es^, induces transcriptional activation of distinct AR target genes [16]. It has been reported that treatment-induced point mutation, W741C, in full length-AR allows bicalutamide to act as an AR agonist [53,55,56]. Additionally, bicalutamide was shown to cause an increase in AR co-activator recruitment to the nucleus as well as an increase in AR expression in the cytoplasm [56]. Increased AR expression in the cytoplasm allows for ligand-binding in the context of low ligand availability, as is the case with patients undergoing ADT. Thus, there are numerous ways that treatment with ADT and bicalutamide can lead to acquired resistance and tumor regrowth.

### 2.2. Currently Targeted Transcription Factors in Castrate Resistant Prostate Cancer (CRPC)

Biochemical recurrence is associated with a rise in Prostate-Specific Antigen (PSA) level in patients after some previous intervention such as surgery, hormonal depletion, or radiation [46]. Patients that have an increased PSA are thus considered to have Castrate Resistant Prostate Cancer (CRPC), also known as hormone refractory PCa. Biochemical recurrence happens for different changes in AR including gene amplification, point mutations, increased expression, increased enzymatic expression of androgen biosynthesis proteins, splice variant upregulation, and increased co-activator recruitment. Modifications of the androgen-signaling pathway and of AR itself are a result of acquired treatment resistance. These changes are clinically used to determine the next course of patient treatment.

#### 2.2.1. Androgen Biosynthesis Inhibitor: Abiraterone

**Figure 7:**
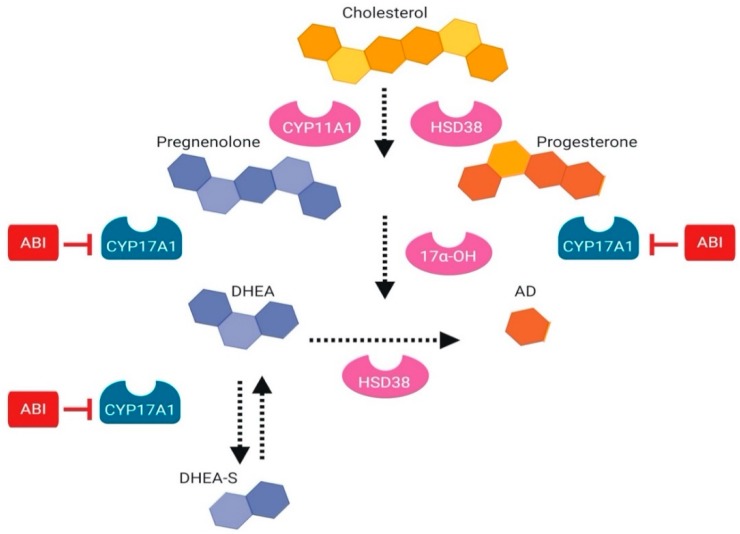
**Schematic illustration of abiraterone inhibition.** Abiraterone inhibits intratumoral synthesis of cholesterol into active androgens that can weakly bind AR in the context of low ligand availability. Abiraterone acts through the enzymatic inhibition of CYP17A1, which ultimately prevents synthesized androgens from further reduction to a more active state. *Abbreviations: ABI, Abiraterone; AD, Androstendione; DHEA, Dehydroepiandrosterone.*

Abiraterone is used as a treatment for CRPC. It indirectly reduces the transcriptional activity of AR through decreasing the ligand availability for receptor activation. Abiraterone is a downstream inhibitor of Cytochrome P450 (CYP17), an enzyme that synthesizes ligands required for nuclear hormone receptors such as PR, AR, and ER [57]. Abiraterone is a competitive inhibitor of the CYP17A enzyme [58]. The binding of abiraterone occurs at the 1720-lyase active site of CYP17A and inhibits re-entrance of steroids in the biosynthesis pathway (Figure 7). This prevents further synthesis and subsequent reduction into a more active state [58].

Treatment with abiraterone has been shown to extend cancer free-survival for 35 months in patients not previously treated with chemotherapy [59]. Inhibition of enzymatic function in the androgen synthesis pathway, rather than directly acting on AR, reduces the chance of AR undergoing point mutations. Additionally, abiraterone inhibits intratumoral biosynthesis of androgens which commonly occurs in patients that had previously received some form of ADT, a known mechanism of AR reactivation [52]. While abiraterone is highly effective at decreasing intratumoral synthesis of androgens and tumor size, it is non-curative [60]. Extended treatment with abiraterone causes an increase in gene and mRNA expression of enzymes involved in androgen biosynthesis [60]. Furthermore, studies in xenograft models indicate that prolonged treatment causes an upregulation of AR^V^567^es^ [16,60]. Upregulation of AR splice variants could be the reason that abiraterone is less effective in patients that have previously undergone ADT. Moreover, abiraterone alone was not shown to be effective at reducing other mechanisms of tumor resistance such as reducing AR splice variant expression [58,61].

#### 2.2.2. Androgen Receptor Antagonists: Enzalutamide, Darolutamide

**Figure 8:**
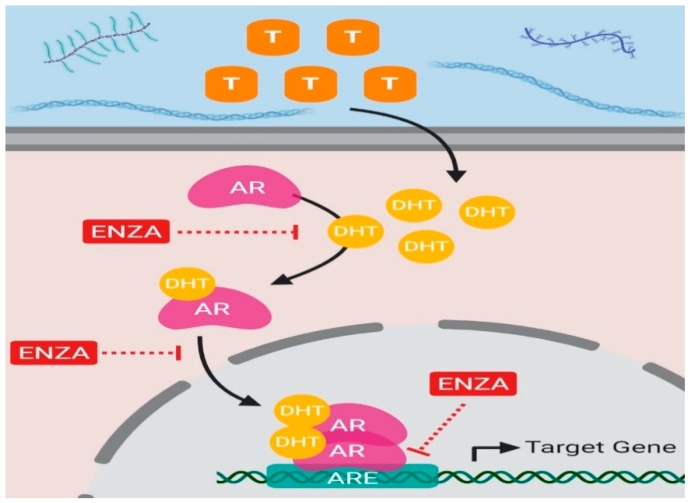
**Schematic illustration of enzalutamide inhibition.** When testosterone enters the cytoplasm of a tissue-specific cell it can get reduced to a more active form known as DHT which acts as a ligand for AR. When DHT binds to AR it undergoes a conformational change and it disassembles from chaperone proteins, HSP90 and HSP70, allowing it to translocate to the nucleus. Once AR translocates to the nucleus it can homodimerize and bind androgen response elements on target gene DNA. Enzalutamide is an AR antagonist that prevents ligand binding at the LBD which inhibits the ability of AR to translocate to the nucleus by inhibiting conformational changes required for AR nuclear translocation and subsequent binding of AR to the DNA of target genes at ARE binding sites. *Abbreviations: T, Testosterone; AR, Androgen Receptor; DHT, Dihydrotestosterone; ARE, Androgen Response Elements; ENZA, Enzalutamide.*

Second-generation AR antagonists, such as enzalutamide and recently FDA-approved compound darolutamide, have been shown to inhibit multiple facets of AR activity, making potent antagonists compared to first-generation agents [62]. All second-generation AR antagonists act as a competitive inhibitor of the androgen receptor (Figure 8).

Enzalutamide was designed to function in a castration-resistant setting wherein there is either gene amplification or increased expression of AR [49]. Compared to first generation anti-androgens, enzalutamide has an increased affinity and selectivity for AR, reducing any chance of off-target effects [49]. Enzalutamide binding to AR induces a conformational change which inhibits DNA binding, nuclear localization, and co-activator binding at the NTD and just outside the AF-2 domain [49]. Enzalutamide binding induces conformational changes within the LBP that decrease recruitment of co-activators. Co-activator recruitment has been shown in other anti-AR agents (i.e., bicalutamide) to play a role in the underlying mechanisms leading to treatment resistance [48,50]. Similarly, changes induced by enzalutamide binding inhibit α-importin from binding the hinge region which contains the nuclear localization signal [5]. Finally, inhibition of DNA binding most likely occurs due to interference with the intramolecular interaction required for zinc finger motif stabilization [1,49].

The main strength of enzalutamide treatment is its effectiveness in decreasing tumor size and cancer progression at a stage of disease that most other treatments are ineffective. Enzalutamide is one of the most potent competitive AR antagonists available and has a relatively high affinity for AR that is 2.5 fold less than that of DHT [49].

Despite all of its favorable attributes, enzalutamide treatment is not curative, as treatment resistance is still acquired. A known mechanism of acquired resistance to enzalutamide treatment is a point mutation F876L in the LBD of full length-AR, which changes enzalutamide function from an antagonist to a partial agonist [63]. This point mutation results in a conformational change that increases the availability of α-helix 12 in the AF-2 region of the LBD of AR for co-activator recruitment [63]. Furthermore, studies have shown that enzalutamide even binds with a high affinity to AR-F876L mutant and furthermore rescues AR target gene expression to promote cell growth and proliferation [63].

In addition, similar studies have also demonstrated that enzalutamide treatment induces expression and activity of the glucocorticoid receptor (GR) in tissues where it would not normally be active [64]. In some instances, GR has inverse effects on a subset of AR target genes, hence where AR would normally activate transcription of a gene, GR represses it. A target gene of AR which is important for repression of prostate cancer progression is Snail Family Transcriptional Repressor 2 (SNAI2), yet its expression is repressed by enzalutamide-treatment induced GR expression [64]. In other instances, GR regulates AR target genes in a like manner. These findings elucidate a possible mechanism of biochemical recurrence and tumor growth in patients undergoing enzalutamide treatment. Furthermore, enzalutamide is designed as a competitive antagonist for full length-AR but fails to antagonize functional AR splice variants. In a disease that expresses AR splice variants, enzalutamide could be selecting a more invasive cancer due to the inability to inhibit splice variant functions [62].

Given the resistance developed to anti-AR treatment like enzalutamide, there have been attempts to develop drugs that target the intrinsically disordered N-terminal transactivation domain of AR which regulates its transcriptional activity. Ralaniten (also known as EPI) was the first small molecule inhibitor that directly binds to an intrinsically disordered transactivation unit (Tau-5) within the AF-1 region in the NTD of AR to enter into clinical trials [65]. The advantage to targeting the NTD is that the LBD where most treatment-induced mutations develop are in the C-terminus. Additionally, these drugs will also be effective against AR splice variants with transcriptional activity. Unfortunately, these drugs have not made it to the clinic yet.

The FDA approved darolutamide in July of 2019 for the treatment of non-metastatic prostate cancer. Darolutamide is also a competitive inhibitor of AR; however, it has a distinct structure with lower blood–brain barrier penetration, which can potentially decrease severe side effects that can present with enzalutamide treatment (e.g., seizures and fractures) [66].

### 2.3. Targeting STAT3 in Androgen-Independent Prostate Cancer

Androgen independent prostate cancer is an advanced stage metastatic CRPC (mCRPC), a terminal disease with limited treatment options. Around 10% of all patients diagnosed with prostate cancer progress into AR independent mCRPC with a two-year survival rate [46,67]. Androgen independent prostate cancer occurs after acquired resistance to enzalutamide treatment or at the stage of disease progression when androgen targeted treatments are no longer effective at reducing tumor burden or disease progression [46]. Thus, identification of alternative pathway dysregulation contributing to PCa progression independent of AR signaling is an active area of prostate cancer research. In this effort, one identified potential target is the Signal transducer and activator of transcription 3 (STAT3).

### 2.4. Use of Antisense Oligonucleotides (ASO) in Androgen-Independent Prostate Cancer

Upregulation of STAT3 is implicated in prostate cancer and specifically in androgen-independent prostate cancers [68]. Antisense oligonucleotide (ASO) targeting STAT3 mRNA has been a promising therapy in the treatment of androgen-independent prostate cancer currently being tested in clinical trials. However, Targeting of STAT3 has been challenging due to the essential role of STAT3 in normal physiological function [69]. Activation of STAT3 requires phosphorylation at Y705 and Y727 by Src kinase and MAP kinase, respectively [70]. Phosphorylation of STAT3 allows for dimerization, nuclear translocation, and subsequent transcriptional activation [70].

An innovative approach to STAT3 targeting is the use of CpG conjugated-STAT3 ASO, which is a bi-functional molecule that utilizes two processes to attack tumors [69]. This molecule is able to activate the immune system and inactivate STAT3 expression [69]. The CpG motifs represent a synthetic modification of cysteine triphosphate and guanine triphosphate bound by a phosphodiester bond and is recognized by the pattern recognition receptor toll-like receptor 9 (TLR-9) expressed on myeloid-derived suppressor cells (MDSC) [69]. The STAT3-ASO component binds to complementary STAT3 mRNA strands, preventing translation of STAT3 [71,72]. The use of CpG conjugated STAT3-ASO in the treatment of androgen-independent prostate cancer would provide a treatment option for a stage of prostate cancer that currently has no treatment. For the first time, CpG-STAT3 ASO has shown promise in reducing the tumor size of localized bone metastasis using in vivo models [69]. Furthermore, the combination of STAT3 inhibitors with a selective target that allows only cells expressing TLR-9 (i.e., MDSC) to be affected, reduces off-target effects that would arise from STAT3 inhibition in other tissues [69].

Although the therapy seems very promising in the future of androgen-sensitive PCa treatment, there are some limits to consider. The treatment with CpG-STAT3 ASO was not effective at reducing localized and distance prostate cancer tumors [69]. Additionally, CpG-STAT3 ASO requires further optimization before use in a clinical setting due to its short half-life, as well as its inability to diffuse through the cell membrane, and the blood–brain barrier [69].

## 3. Conclusions

Many nuclear receptor superfamily members have been implicated in cancer development. Currently, around 10% of FDA approved drugs for the treatment of human malignancies target nuclear hormone receptors [44]. This review focuses on clinically relevant practices that are currently used for prostate cancer at each stage of disease progression with an emphasis on the treatments that target transcription factors. While numerous features of PCa progression have been linked to AR-targeted treatment induced changes, the identification of markers that allow for earlier detection can help extend patient survival. Although a lot of progress has been made in understanding prostate cancer progression, there are still gaps regarding how androgen and other hormone receptors play a role in this disease. Further understanding of involvement of alternative pathways, such as STAT signaling, can provide new potential and more effective targets for future treatment development.
